# Efficacy of transanal tube placement after anterior resection for rectal cancer: a systematic review and meta-analysis

**DOI:** 10.1186/s12957-016-0854-0

**Published:** 2016-03-31

**Authors:** Shuanhu Wang, Zongbing Zhang, Mulin Liu, Shiqing Li, Congqiao Jiang

**Affiliations:** Department of Gastrointestinal surgery, The First Affiliated Hospital of Bengbu Medical College, Bengbu, Anhui Province China

**Keywords:** Rectal cancer, Transanal tube, Anastomotic leakage

## Abstract

**Background:**

Anastomotic leakage is a serious complication that can occur after anterior resection of the rectum. There is a question regarding whether the placement of a transanal tube can decrease the rate of anastomotic leakage. The aim of this systematic review and meta-analysis was to evaluate the efficacy of transanal tube placement after anterior resection.

**Methods:**

We searched three major databases (PubMed, Embase, and the Cochrane Library) up until January 2015 for studies evaluating the benefit of transanal tubes after anterior resection for rectal cancer. The primary outcome measure was the rate of clinical anastomotic leakage. Secondary outcome was the rate of reoperation. Pooled risk ratios (RR) with 95 % confidence intervals (CI) were obtained using random effects models.

**Results:**

One randomized controlled trial and three observational studies involving 909 patients met inclusion criteria. Clinical anastomotic leakage occurred in 3.49 % (14 of 401) of patients with transanal tubes and 12.01 % (61 of 508) of patients without transanal tubes. Meta-analysis of the studies showed a lower risk of anastomotic leakage (RR, 0.32; 95 % CI 0.18–0.58) and reoperation related to leakage (RR, 0.19; 95 % CI 0.08–0.46) when the transanal tube was placed.

**Conclusions:**

While studies are few and mostly observational, the data to date indicate that placement of a transanal tube decreases the rate of clinical anastomotic leakage and reoperation related to leakage. More studies are needed to confirm these findings.

## Background

In 1908, William Ernest Miles introduced the basis of modern rectal cancer surgery in a study published in Lancet. As late as the past few decades, his abdominoperineal resection has been the gold standard of rectal cancer treatment. In 1948, CF Dixon proposed another technique, one that allowed for sphincter preservation [1]. With the popularization of the concept of total mesorectal excision (TME) and improvements in surgical instruments, low anterior resection has become more commonly performed than permanent stoma. With the increase of anus-preserving operations, anastomotic leakage has drawn the attention of surgeons.

Anastomotic leakage is defined as a defect of the intestinal wall at the site of anastomosis (including suture and staple lines in neorectal reservoirs) leading to a communication between the intra- and extraluminal compartments [2]. Anastomotic leakage after anterior resection of the rectum can be serious. The overall frequency of this complication has been reported at 8.58 % [3]. Patients suffering from anastomotic leakage not only remain hospitalized longer but also have a lower survival rate [4, 5]. Low anastomosis and male gender are considered independent risk factors for symptomatic anastomotic leakage [6, 7]. For this reason, surgeons have explored ways of reducing the incidence of anastomotic leakage. Some systematic reviews and meta-analyses have indicated that proximal fecal diversion can reduce the rate of anastomotic leakage and reoperation related to leakage [8–11]. Proximal fecal diversion by loop ileostomy or colostomy requires another surgery to close the stoma. This increases hospital costs and the rate of stoma-related complications [12]. While some surgeons place a defunctioning transanal tube in an attempt to reduce anastomotic leakage [13], others believe that transanal tube placement is ineffective in preventing leakage [14]. Thus, although defunctioning transanal tube placement is widely used in anterior resection for rectal cancer, it remains unclear whether this measure is useful to patients. We conducted this systematic review and meta-analysis of available data to determine whether a transanal tube reduces postoperative complications in patients undergoing anterior resection for rectal cancer.

## Methods

### Publication search

The electronic databases Pubmed, Embase, and the Cochrane Library were searched by two authors (S.W. and Z.Z.) up till January 2015. The search strategy included the following keywords in various combinations: “transanal tube,” “transanal drainage,” “transanal drainage tube,” “transanal catheter,” “anterior resection,” “rectal cancer,” and “anastomotic leakage.” Free text searches and MeSH searches were performed. Of the articles included here, references were read to identify any related articles. No language restrictions were applied.

The inclusion criteria were biopsy-proven rectal cancer before operation, laparotomy or laparoscopy, radical resection, use of stapler anastomosis, and comparison of anterior resection with a transanal tube to anterior resection without a transanal tube. The exclusion criteria were defunctioning stoma, defunctioning stoma and transanal tube placement at the same time, emergency operation, and palliative operation.

### Data extraction

Two authors independently assessed all titles and abstracts for relevance (S.W. and S.L.). Disagreements were resolved through discussion. In cases where no consensus could be reached, a third specialist was consulted (C.J.). If a study covered both handsewn and stapler anastomosis, it was included only if a breakdown of data by level of anastomosis was available. Studies were excluded if all anterior resections (both curative and palliative) were included, without any breakdown by level of anastomosis.

Two outcome variables were evaluated: clinical anastomotic leakage (primary outcome) and reoperation related to leakage (secondary outcome). Clinical anastomotic leakage was defined as the presence of clinical symptoms such as peritonitis, fever, or septicemia combined with the occurrence of pelvic abscess, discharge of feces, pus, or gas from the drainage tube, and formation of a rectovaginal fistula [15, 16]. Radiologically confirmed anastomotic leakage with no clinical signs was not included. When the required information could not be obtained from the article, e-mails were sent to the authors requesting it. If there was no reply from the author, the data were considered missing.

Two authors (S.W. and M.L.) assessed the quality of the included articles. The Jadad scoring system was used to assess the quality of RCT [17]. The quality of the observational studies was assessed using the Newcastle-Ottawa quality assessment scale [18].

### Statistical analysis

Statistical analysis was performed using Review Manager (RevMan, version 5.3, The Nordic Cochrane Centre, the Cochrane Collaboration, Copenhagen, Denmark). Statistical heterogeneity was assessed using *I*^*2*^ and *χ*^2^ statistics. We estimated pooled risk ratio (RR) and 95 % confidence interval (CI) for each outcome. Heterogeneity was considered significant if the *P* value (*χ*^2^) was <0.1 or *I*^*2*^ was >50 %. A random effects model was used even if no significant heterogeneity statistical heterogeneity was noted. This takes into account the low statistical power of tests of heterogeneity and the likelihood that clinical heterogeneity may exist even if statistical heterogeneity cannot be demonstrated. Sensitivity analysis was conducted by omitting each study one at a time in order to assess the influence of each single study on the overall risk estimate. Because of the limited number (below 10) of studies included in each analysis, publication bias was not assessed.

## Results

**Fig. 1:**
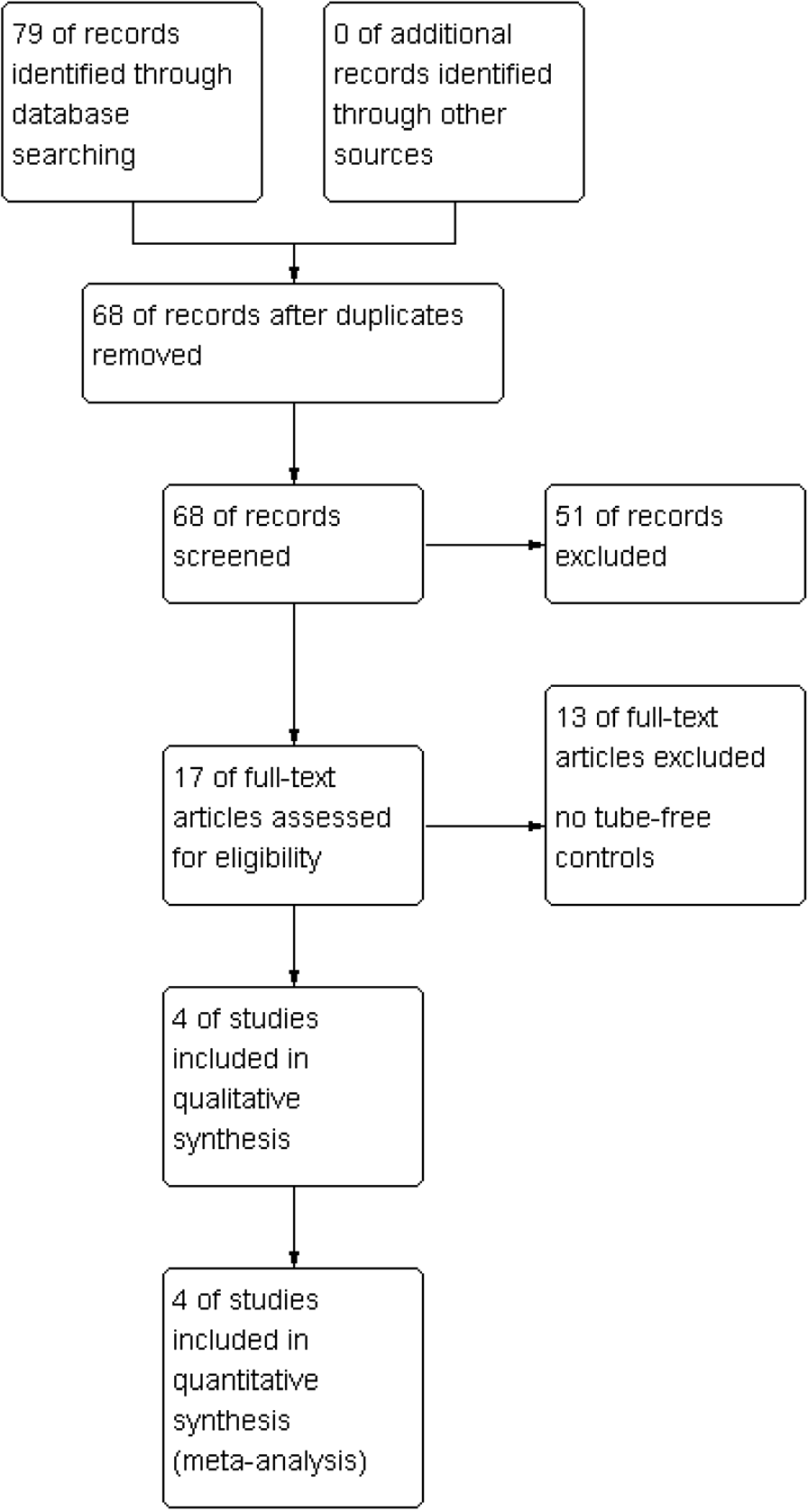
Flow chart of study screening and selection

The initial search retrieved 79 studies. After removal of 11 repeated studies, 68 articles remained. After reading the titles and abstracts of the studies, 51 studies were excluded because 1 was a systematic review (Poster Abstracts in Colorectal Disease) and another 50 were not relevant. Upon further review, 13 studies were excluded because the data regarded the placement of a transanal tube and nothing else. Finally, four articles were included (Fig. 1). The included studies were published between 2011 and 2014. Sample size of studies varied from 158 to 370 patients. One randomized controlled trial [19] and three observational studies [20–22] involved 909 patients, 401 of whom had a transanal tube and 508 did not. All studies reported clinical anastomotic leakage and reoperation related to leakage. Characteristics of the studies included are given in Table 1.

**Table 1:** Study characteristics

Study	Year	No. of patients	Type of operation	*n*		Anastomotic leakage		Reoperation	
				TT	NTT	TT	NTT	TT	NTT
Xiao et al. [19]	2011	370	Laparotomy	188	182	7 (3.7)	17 (9.3)	2 (1.1)	14 (7.7)
Nishigori et al. [20]	2014	176	Laparotomy and laparoscopy	36	140	1 (2.8)	22 (15.7)	1 (2.8)	11 (7.9)
Hidaka et al. [21]	2014	205	Laparoscopy	96	109	4 (4.2)	15 (13.8)	0 (0)	10 (9.2)
Zhao et al. [22]	2013	158	Laparotomy	81	77	2 (2.5)	7 (9.1)	2 (2.5)	7 (9.1)

Data are presented as *n* (%)

*TT* transanal tube, *NTT* non-transanal tube

Three items (randomization, blinding, withdrawals, and dropouts) in the Jadad scoring system were used to assess the quality of the study. Eight elements in Newcastle-Ottawa quality assessment scale were used to assess patient population and selection, study comparability, follow-up, and outcome of interest. High-quality elements are awarded by adding a star, and then the stars are added up to compare the study quality. Assessment of the methodologic quality of studies using the three items in the Jadad scoring system for randomized trials and eight items in the Ottawa quality assessment scale for observational studies are shown in Table 2.

**Table 2:** Jadad score and Newcastle-Ottawa quality assessment scale

Study	Jadad score (0–5)	Newcastle-Ottawa quality assessment scale cohort studies			
		Selection (0–4)	Comparability (0–2)	Outcome (0–3)	Total (0–9)
Xiao et al. [19]	2	****	**	***	9
Nishigori et al. [20]	–	****	*	***	8
Hidaka et al. [21]	–	****	*	***	8
Zhao et al. [22]	–	****	**	***	9

There is one star for each point, a study can be given a maximum of nine stars

**Fig. 2:**
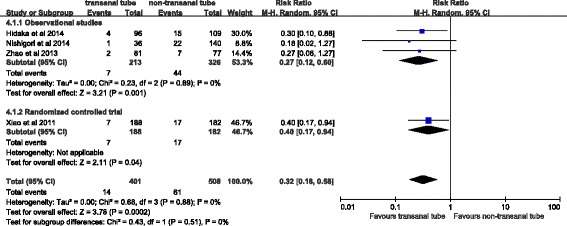
Forest plot of the rate of clinical leakage

**Fig. 3:**
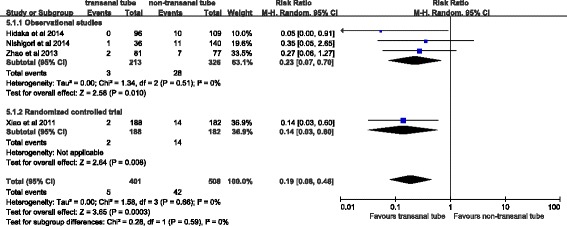
Forest plot of reoperation rate

Four studies reported on clinical anastomotic leakage and reoperation. The pooled results from the three observational studies showed that transanal tubes was associated with a lower risk of both anastomotic leakage (pooled RR 0.32, 95 % CI 0.18–0.58, *P* = 0.0002, Fig. 2) and reoperation (0.19, 95 % CI 0.08–0.46, *P* = 0.0003, Fig. 3). There was no significant heterogeneity for either outcome. The pooled RR for the three observational studies was similar to the RR in the randomized trial for both anastomotic leakage (0.27, 95 % CI 0.12–0.60 versus 0.40, 95 % CI 0.17–0.94) and reoperation (0.23, 95 % CI 0.07–0.70 versus 0.14, 95 % CI 0.03–0.60). Sensitivity analysis was performed by excluding each single study at a time. The effect on anastomotic leakage did not materially alter the pooled RR, which ranged from 0.27 (95 % CI 0.12–0.60, *P* = 0.001) to 0.34 (95 % CI 0.19–0.63, *P* = 0.0006). The pooled RR for reoperation ranged from 0.16 (95 % CI 0.05–0.47, *P* = 0.001) to 0.23 (95 % CI 0.07–0.70, *P* = 0.01).

## Discussion

Anastomotic leakage is a serious complication following surgery for rectal cancer. With the development of surgical instruments and surgical techniques, sphincter-preserving procedures have become more prevalent [23]. However, the risk of anastomotic leakage is increasing [24, 25], and placement of a transanal tube for its prevention is controversial.

The present meta-analysis was conducted to evaluate existing data to help clarify the role of transanal tube placement in the prevention of anastomotic leakage and reoperation. Results of both the meta-analysis of the three observational studies and the one randomized trials indicate that placement of a transanal tube significantly decreases the risk of leakage and reoperation. While the mechanism of action of the transanal tube is unclear, it may be related to reduction in endoluminal pressure in the anastomotic portion of the intestine which may be an important factor in anastomotic leakage [26]. This is supported by the observation that rectal resting pressure was lower in the transanal tube group than in the tube-free group in one study [19]. The transanal tube may also directly drainage on the proximal side of the anastomosis [27, 28]. If anastomotic leakage occurs, a large amount of stool will leak to the peritoneal cavity in the tube-free group. Reoperation for anastomotic leakage will be inevitable. The rate of reoperation for anastomotic leakage was 8.3 % (42 of 508) in the tube-free group. In contrast, stool could be drained from the rectum through the transanal tube. A small amount of stool might leak to the peritoneal cavity resulting in localized peritonitis. The localized peritonitis was cured conservatively by the placement of abdominal drainage tube. So the rate of reoperation for anastomotic leakage was only 1.3 % (5 of 401) in the tube group.

These results are similar to those published in an earlier meta-analysis by Shigeta and co-workers [29]. However, that study was published only in a poster abstract. No further details were made available. The purpose of the present article is to fill this gap.

This meta-analysis followed clear methodology with clearly predefined inclusion and exclusion criteria, outcome measures, study quality appraisal, and statistical methods a priori. However, there are limitations that should be considered. First, there was only one RCT and three observational studies available for inclusion. Second, among the included studies, some patients underwent laparotomy and others underwent laparoscopy. But the rate of anastomotic leakage was not significant differences between laparotomy and laparoscopy group [30, 31]. The outer diameters of the transanal tube also varied from 24 to 28 Fr. These differences constitute clinical heterogeneity even though statistical heterogeneity was not demonstrated. Finally, the sample size of all studies was relatively small. However, the sensitivity of the analysis indicated that the results were robust.

Although transanal tube may reduce the risk of anastomotic leakage and reoperation, its placement increases patients’ discomfort and inconvenience. There have also been reports of tubes perforating bowel especially in the region anterior to the sacrum [20]. However, compared with the reoperations required for anastomotic leakage in the absence of a transanal tube, complications associated with a transanal tube are relatively minor.

## Conclusions

This systematic review and meta-analysis indicates that placement of transanal tubes after anterior resection reduces the risk of anastomotic leakage and reoperation. However, the studies are few and mostly observational. A well-powered, multicenter, randomized, controlled trial is needed to confirm these findings. If confirmed, the use of this intervention will improve outcomes and reduce complications in patients undergoing anterior resection.

## Abbreviations

NTT: non-transanal tube
TME: total mesorectal excision
TT: transanal tube
